# Supplementary figures and images for: Diaphorobacter nitroreducens synergize with oxaliplatin to reduce tumor burden in mice with lung adenocarcinoma
Source: mSystems. 2024 Mar 14;9(4):e01323-23. doi: 10.1128/msystems.01323-23 (PMC11019951; doi:10.1128/msystems.01323-23)

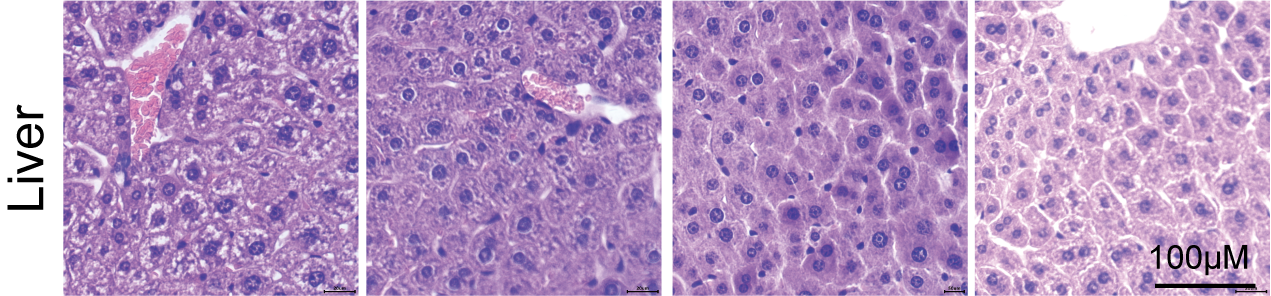

Supplement: Figure S1 — H&E staining of the liver (400×). [file msystems.01323-23-s0001.tif]

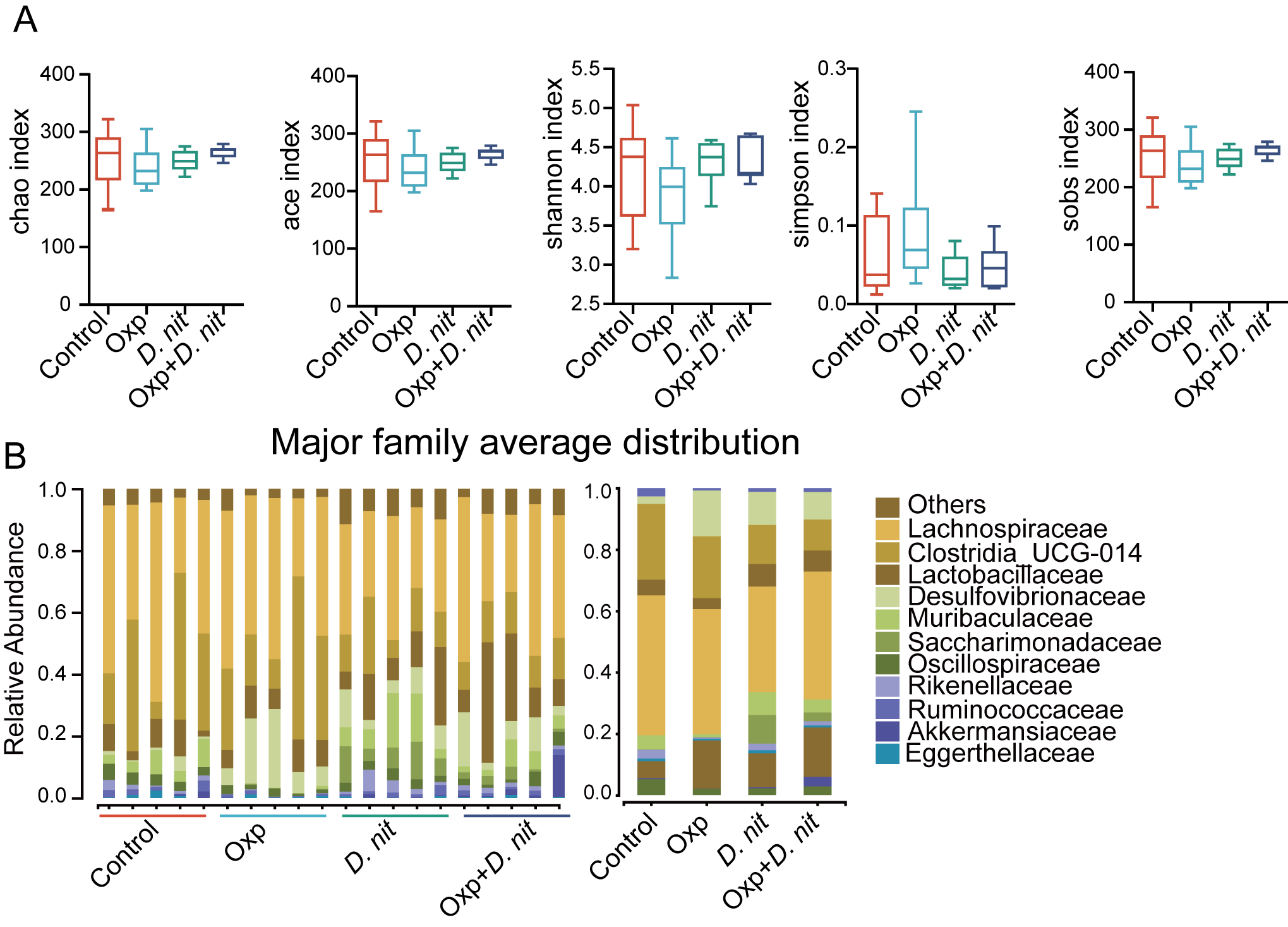

Supplement: Figure S2 — (A) Alpha diversity analysis including Chao, Ace, Shannon, Simpson, and Sobs indices. (B) Bar plots showing the composition and relative abundance of each sample (left) and mean relative abundance in control, Oxp, D. nit, and Oxp+ D. nit groups (right) at the family level. [file msystems.01323-23-s0002.tif]

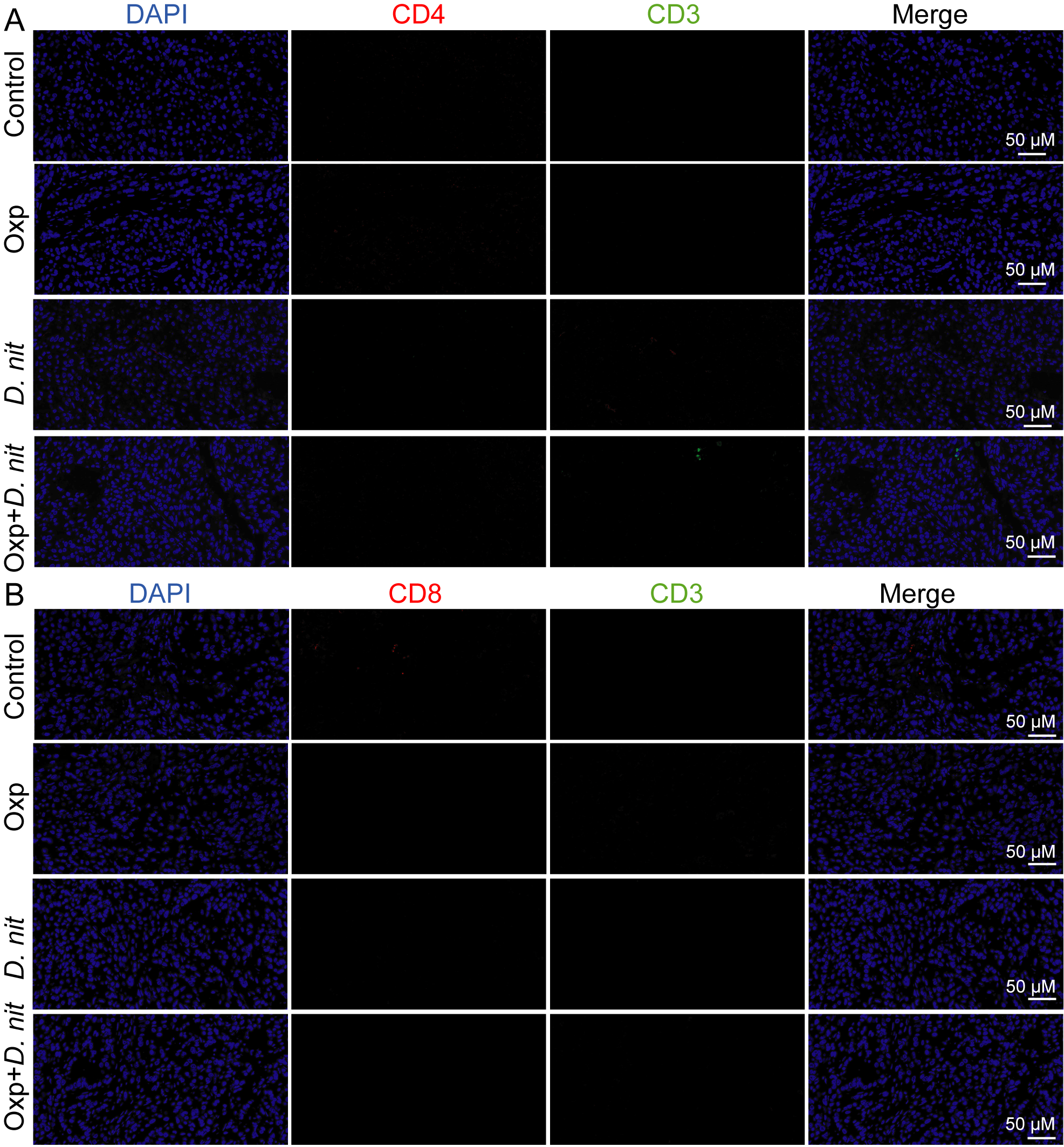

Supplement: Figure S3 — (A) Immunofluorescence staining of the helper T cell markers CD4 (pink) and CD3 (green) with DAPI nuclear staining (blue), 40×. (B) Immunofluorescence staining of cytotoxic T cell markers CD8 (pink) and CD3 (green) with DAPI nuclear staining (blue), 40×. [file msystems.01323-23-s0003.tif]
